# MicroRNA-182-5p protects human lens epithelial cells against oxidative stress-induced apoptosis by inhibiting NOX4 and p38 MAPK signalling

**DOI:** 10.1186/s12886-020-01489-8

**Published:** 2020-06-17

**Authors:** Zhao-Na Li, Ming-Xu Ge, Zhong-Fang Yuan

**Affiliations:** 1 Department of Ophthalmology, The second people’s Hospital of Jinan, Jinan, Shandong 250001 People’s Republic of China; 2 Department of Neurosurgery, Shandong Provincial Hospital, Jinan, Shandong 250021 People’s Republic of China; 3 Department of Ophthalmology, Jinan Central Hospital, Affiliate of Shandong University, Jinan, Shandong 250013 People’s Republic of China

**Keywords:** Cataract, Oxidative stress, miR-182-5p, NOX4

## Abstract

**Background:**

MicroRNAs (miRNAs) are abnormally expressed in various ocular diseases, including age-related cataract. However, the role of miR-182-5p in the progression of age-related cataract remains unclear.

**Methods:**

The expression of miR-182-5p in HLE-B3 cells was detected by qRT-PCR. HLE-B3 cells were transfected with miR-182-5p mimics. CCK-8, EdU, flow cytometry, 2′,7′-dichlorodihydrofluorescein diacetate, JC-1 kit, and western blot were used to assess the cell viability, proliferation, apoptosis, reactive oxygen species (ROS) level, mitochondrial membrane potential (MMP), and protein expression, respectively, in vitro. The relationship between miR-182-5p and NOX4 was confirmed using the dual-luciferase reporter gene analysis.

**Results:**

We found that miR-182-5p expression was significantly decreased by the H_2_O_2_ exposure. Overexpression of miR-182-5p promoted cell proliferation and inhibited ROS production and apoptosis in H_2_O_2_-induced HLE-B3 cells. Moreover, p-p-38, p-ERK, and p-JNK were up-regulated in H_2_O_2_-treated HLE-B3 cells, and overexpression of miR-182-5p reversed the effects of H_2_O_2_ on HLE-B3 cells. In addition, dual-luciferase reporter assay substantiated that NOX4 was a direct target and downregulated by miR-182-5p.

**Conclusions:**

We concluded that miR-182-5p inhibited lens epithelial cells apoptosis through regulating NOX4 and p38 MAPK signaling, providing a novel biomarker for treatment of age-related cataract.

## Background

Cataract is characterized by progressive opacity of the ocular lens, which can lead to blindness [1]. Approximately 50% of the blindness in middle-income and low-income countries is caused by cataracts [2]. Until now, multiple risk factors like aging, diabetes, genetics, oxidative stress and UV exposure have been associated with the pathogenesis of age-related cataract [3]. Although cataract removal and intraocular lens implantation surgery are effective procedures, letting patients see the light again [4]. However, there are disadvantages in replacing tissues and organs with artificial materials. Surgery may result in severe postoperative complications, including wound leakage, corneal abrasion, and ocular hypertension, especially in the elderly [5]. The number of age-related cataract cases increases from 35.77 million in 1990 to 79.04 million in 2015. It is projected that, by 2050, the number of age-related cataract cases will reach 187.26 million in China [6]. Owing to the prevalence of the disease among ageing populations, cataract surgeries amount to a significant proportion of healthcare costs, especially in remote and poor areas of developing countries [2]. Therefore, in-depth study of the pathogenesis of age-related cataracts by preventing the occurrence of cataracts or delaying their development has become a promising area of research.

Oxidative damage to the human lens epithelial cells (LECs) is one of the major factors leading to apoptosis which is considered as an early event of cataract development [7, 8]. MicroRNAs (miRNAs) are single-stranded, short, non-coding molecules that have vital roles in the negative regulation of target genes, leading to the repression of the translation process [9]. MiRNAs are involved in numerous fundamental cellular processes, including cell differentiation, proliferation and apoptosis. MiR-182 (miR-182-5p) is reported to play an important role in ophthalmic disorders, including pterygium [10], high-tension glaucoma [11], congenital cataract [12], retinoblastoma [13], and macular degeneration [14]. However, the exact role of miR-182-5p in the progression of age-related cataract and the underlying mechanism remain poorly understood.

In the present study, we measured the expression of miR-182-5p in LECs upon exposure to H_2_O_2_ and explored that miR-182-5p suppressed LECs apoptosis by regulating the nicotinamide adenine dinucleotide phosphate oxidase subunit 4 (NOX4) and p38 mitogen-activated protein kinase (MAPK) signalling.

## Methods

### Cell culture

Human lens epithelial B3 (HLE-B3) cells were obtained from American Type Culture Collection (ATCC, Rockville, MD, USA). Cells were cultured in Eagle’s minimum essential medium (EMEM; Gibco, Carlsbad, CA, USA) supplemented with 10% fetal bovine serum (FBS; Gibco) at 37 °C in a humidified chamber with 5% CO_2_.

### Cell transfection

MiR-182-5p mimics or negative controls (RiboBio, Guangzhou, China) were transfected into HLE-B3 cells using the Lipofectamine 3000 reagent (Invitrogen, Carlsbad, CA, USA) following the manufacturer’s instructions. HLEC-B3 cells were treated with pcDNA3.1-NOX4 (oe-NOX4) or pcDNA3.1 negative control (oe-NC) (RiboBio, Guangzhou, China), followed by treatment with miR-182-5p mimics or negative controls. At 48 h post transfection, HLE-B3 cells were treated with H_2_O_2_ (250 μmol/L) for 12 h.

### Luciferase assays

The putative binding sites of miR-182-5p and NOX4 were predicted by TargetscanHuman 7.2. The 3′untranslated regions (3′UTR) sequences containing wild-type or mutant binding sites of NOX4 were subcloned into pmirGlO luciferase reporter vector (Promega, Madison, WI, USA) to generate the wild-type (NOX4-WT) or mutant-type plasmids (NOX4-MUT), respectively. The miR-NC or miR-182-5p mimics were cotransfected with reporter plasmids into HLE-B3 cells using Lipofectamine 3000. Luciferase activities were analyzed 24 h after transfection using the Dual-luciferase Reporter Assay Kit (Promega, Madison, USA).

### Cell counting kit-8 (CCK-8) assay

Cells were seeded in a 96-well plate (1 × 10^4^). At 24, 48, 72 and 96 h, 10 μL of CCK8 reagent (Beyotime Institute of Biotechnology, Jiangsu, China) was added to the cells. The absorbance of the wells was measured at 450 nm using a microplate reader (Bio-Tek, Winooski, VT, USA).

### 5-Ethynyl-2′-deoxyuridine (EdU) assay

To investigate the influence of miR-182-5p on cell proliferation, EdU proliferation assay (RiboBio, Guangzhou, China) was conducted. Briefly, cells were incubated with 50 μM EdU for 2 h at 37 °C. Cells were fixed with 4% paraformaldehyde and treated with 0.5% Triton X-100 at room temperature. Next, the cells were washed with phosphate buffered saline (PBS) and incubated with Hoechst 33342 (100 μL) at room temperature for 30 min. The EdU positive cells were then visualized under a fluorescence microscope (Leica, Germany).

### Apoptosis detection

Cellular apoptosis was determined by flow cytometry using the Annexin V-fluorescein isothiocyanate (V-FITC)/propidium iodide (PI) kit (KeyGEN Biotech, Nanjing, China). Briefly, the collected cells were resuspended in 500 μL of 1× binding buffer, 5 μL Annexin V-FITC and 5 μL PI were added and incubated at room temperature in the dark for 15 min. Cell apoptosis was analyzed by using a flow cytometer (A60-Micro, Apogee, UK).

### Detection of mitochondrial membrane potential (MMP)

Cells were added to 6-well plates (1 × 10^6^) and divided into groups as described for cell transfection. The changes of cell MMP in different groups of cells were measured using 5 μg/mL JC-1 (Beyotime Biotechnology, Shanghai, China). The cells were washed with PBS and detected by flow cytometer (Apogee, UK).

### Detection of oxidative stress products

The concentrations of reactive oxygen species (ROS) in the cells were measured by adding 200 μL 2′-7′-dichlorofluorescin diacetate (DCFH-DA) (5 μmol/L final concentration, Sigma-Aldrich, St. Louis, MO, USA). After washing, cells were detected by the flow cytometer (Apogee, UK). The malondialdehyde (MDA) contentand superoxide dismutase (SOD) and glutathione peroxidase (GSH-Px) activities were detected using measurement kits (Nanjing Jiancheng Bioengineering Institute, Nanjing, China), separately.

### Quantitative real-time PCR (qRT-PCR)

Total RNA was isolated from LECs using TRIzol reagent. 1 μg RNA was used to reverse transcript to cDNA by using PrimeScript RT Master Mix (TaKaRa, Japan). For qRT-PCR, the SYBR (Roche, Basel, Switzerland) was used according to the manufacturer’s protocol with the Analytik-jena qTOWER PCR System (Jena, Germany). U6 and β-actin were used as an internal control for miR-182-5p and NOX4, respectively. Primers are listed as follows, miR-182-5p (ACACTCCAGCTGGGTTTGGCAATGGTAGAACT and TGGTGTCGTGGAGTCG), U6 (CTCGCTTCGGCAGCACA and AACGCTTCACGAATTTGCGT), NOX4 (CGATTCCGGGATTTGCTACTG and CCTCAAATGGGCTTCCAAATG), β-actin (TGAGCGCGGCTACAGCTT and TCCTTAATGTCACGCACGATTT).

### Western blot

Cells were lysed in lysis buffer to extract protein samples. Total proteins were quantified using the bicinchoninic acid method (Wuhan Boster Biological Technology., LTD, China). 50 μg of total protein was separated by sodium dodecyl sulfate-polyacrylamide gel electrophoresis and transferred onto polyvinylidene fluoride membranes (Millipore, USA). The membranes were probed with appropriate primary antibodies, including Cleaved caspase 3 (#9664, CST, 1:1000), Cleaved caspase 9 (#9509, CST, 1:1000), p-p38 (#4511, CST, 1:1000), p38 (#8690, CST, 1:1000), p-ERK (#4370, CST, 1:1000), ERK (#4695, CST, 1:1000), p-JNK (#9255, CST, 1:1000), JNK (#9252, CST, 1:1000), NOX4 (ab133303, abcam, 1:1000), and β-actin (#3700, CST, 1:5000). Then, membranes were incubated with secondary antibodies (horseradish peroxidase-labeled goat anti-rabbit IgG, ab6721, abcam, 1:10000) for 2 h. Finally, the protein bands were detected by chemiluminescence reagents (Pierce, Rockford, IL, USA).

### Statistical analysis

GraphPad Prism 7 (GraphPad, San Diego, CA, USA) was applied for statistical analysis. All experiments were repeated three times. Data have been presented as the mean ± SD. Differences between multiple groups were assessed by one-way ANOVA and Tukey’s multiple comparisons test. Differences between groups were considered significant when P < 0.05.

## Result

### Overexpression of miR-182-5p alleviates H_2_O_2_-induced inhibition of cell proliferation

**Fig. 1:**
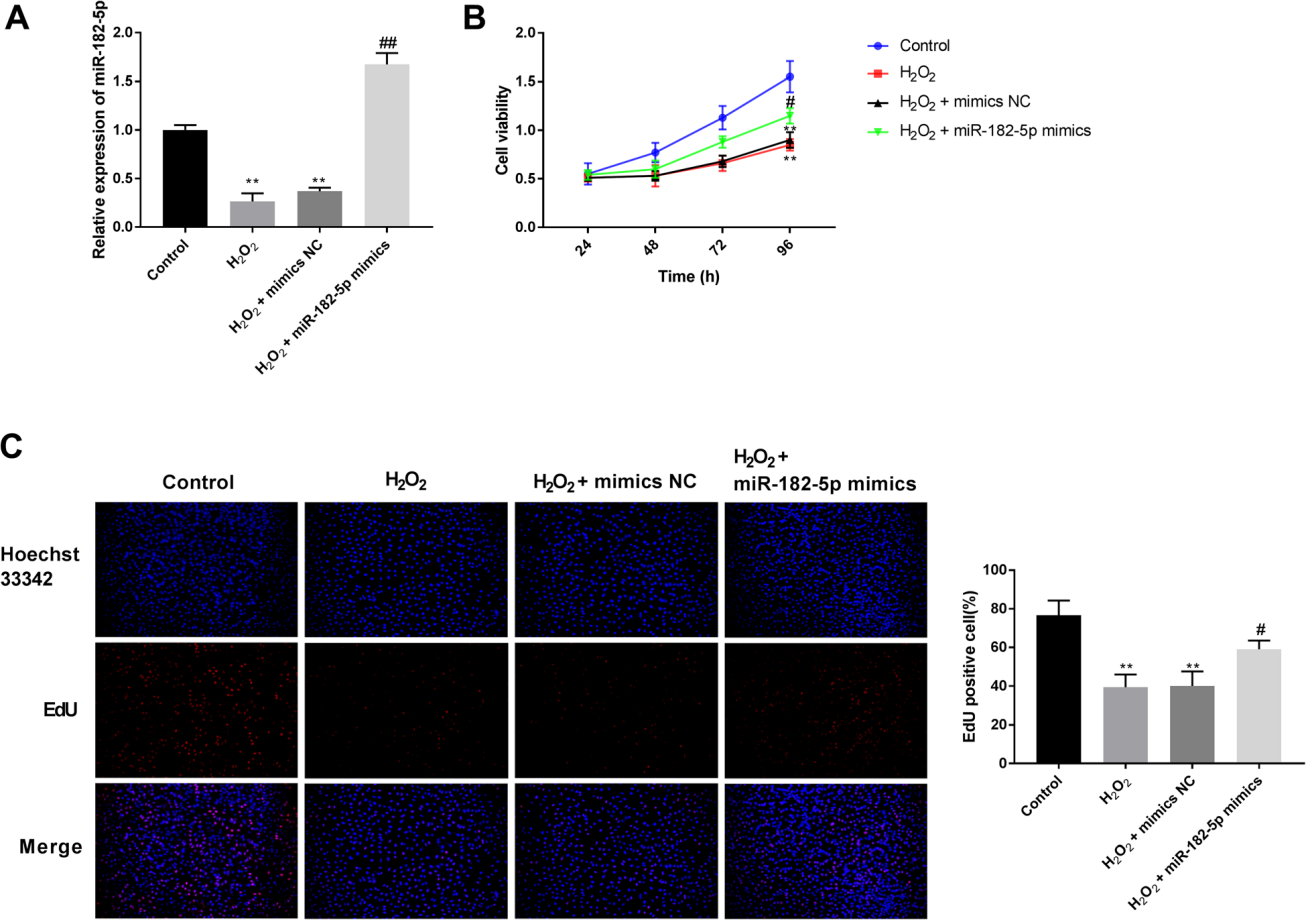
Overexpression of miR-182-5p alleviates H_2_O_2_^−^induced inhibition of cell proliferation. A. Expression of miR-182-5p was detected by qRT-PCR. B. HLE-B3 cell viability was detected by cell counting kit-8 assay. C. HLE-B3 cell proliferation was detected by EdU assay. ** P < 0.01 vs. control group, # P < 0.05 vs. H_2_O_2_ group, ## P < 0.01 vs. H_2_O_2_ group. Independent experiments were carried out three times for each assay

As detected by qRT-PCR (Fig. 1A), miR-182-5p expression was decreased by H_2_O_2_ in HLE-B3 cells. Cells transfected with miR-182-5p mimics exhibited higher miR-182-5p expression, indicating a high transfection efficiency. CCK-8 analysis revealed that H_2_O_2_ treatment greatly decreased the viability of HLE-B3 cells at 48, 72, and 96 h; and transfection with miR-182-5p mimics partially attenuated the cell viability inhibition (Fig. 1B). EdU analysis showed that H_2_O_2_ treatment triggered the proliferative inhibition of HLE-B3 cells, and transfection with miR-182-5p mimics partially attenuated the proliferative inhibition (Fig. 1C).

### Overexpression of miR-182-5p suppresses H_2_O_2_-induced oxidative stress

**Fig. 2:**
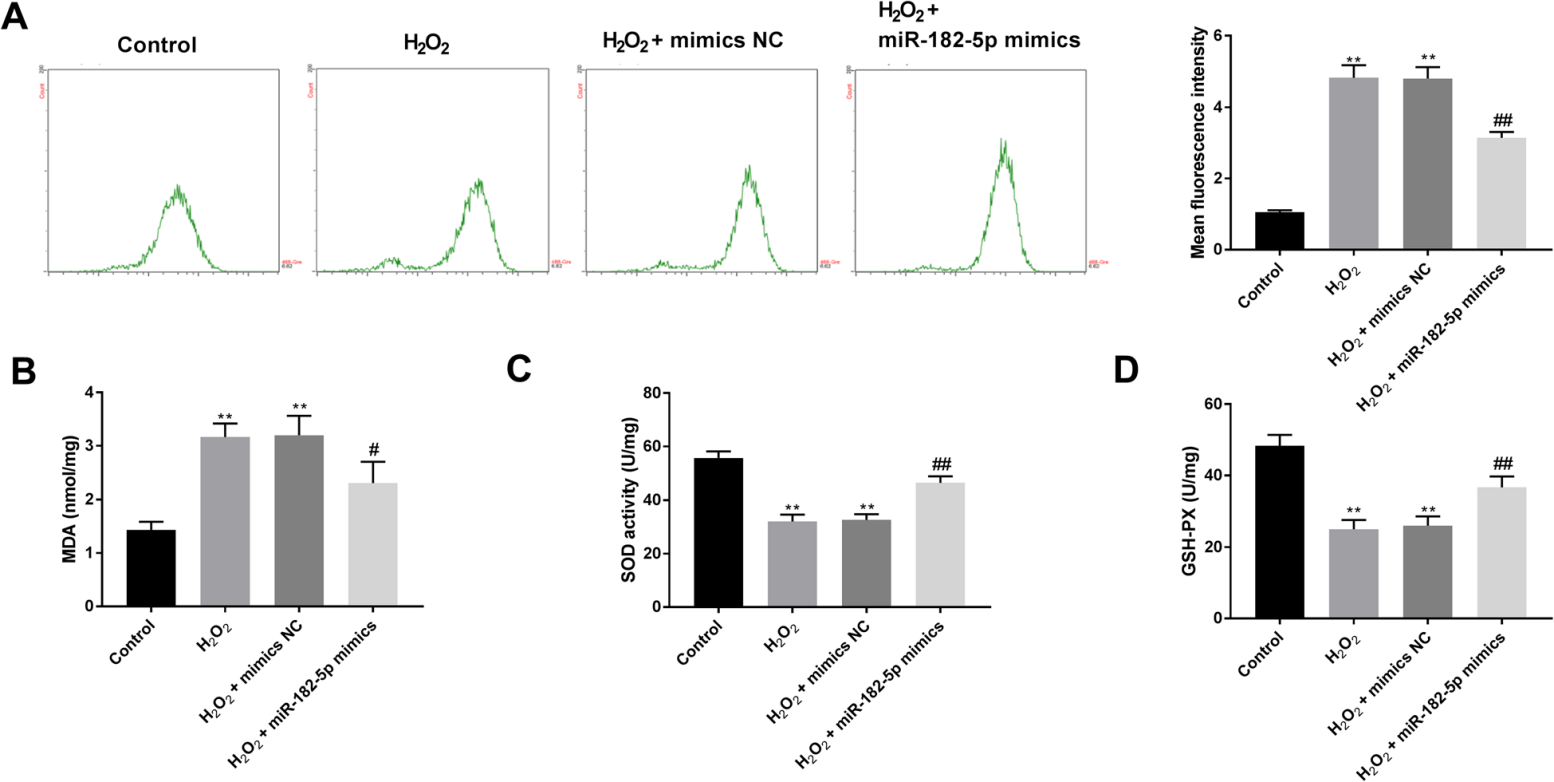
Overexpression of miR-182-5p suppresses H_2_O_2_-induced oxidative stress. A. MFI of ROS in HLE-B3 cells was determined by DCFH-DA assay. B-D. Effect of miR-182-5p on MDA content (B), SOD (C) and GSH-Px (D) activities in HLE-B3 cells. ** P < 0.01 vs. control group, # P < 0.05 vs. H_2_O_2_ group, ## P < 0.01 vs. H_2_O_2_ group. Independent experiments were carried out three times for each assay

The intracellular ROS levels were presented as the mean fluorescent intensity (MFI), as performed by the DCFH-DA method (Fig. 2A). The MFI of intracellular ROS was increased in HLE-B3 cells following H_2_O_2_ treament, but was significantly decreased after transfection with miR-182-5p mimics. Similarly, a higher level of MDA was observed in the H_2_O_2_ group as compared to the control group, this level was significantly decreased after transfection with miR-182-5p mimics (Fig. 2B). In addition, H_2_O_2_ inhibited the activities of SOD and GSH-Px in HLE-B3 cells, which could be reversed by transfection of miR-182-5p mimics (Fig. 2C and D).

### Overexpression of miR-182-5p protects HLE-B3 cells against oxidative stress-induced apoptosis

**Fig. 3:**
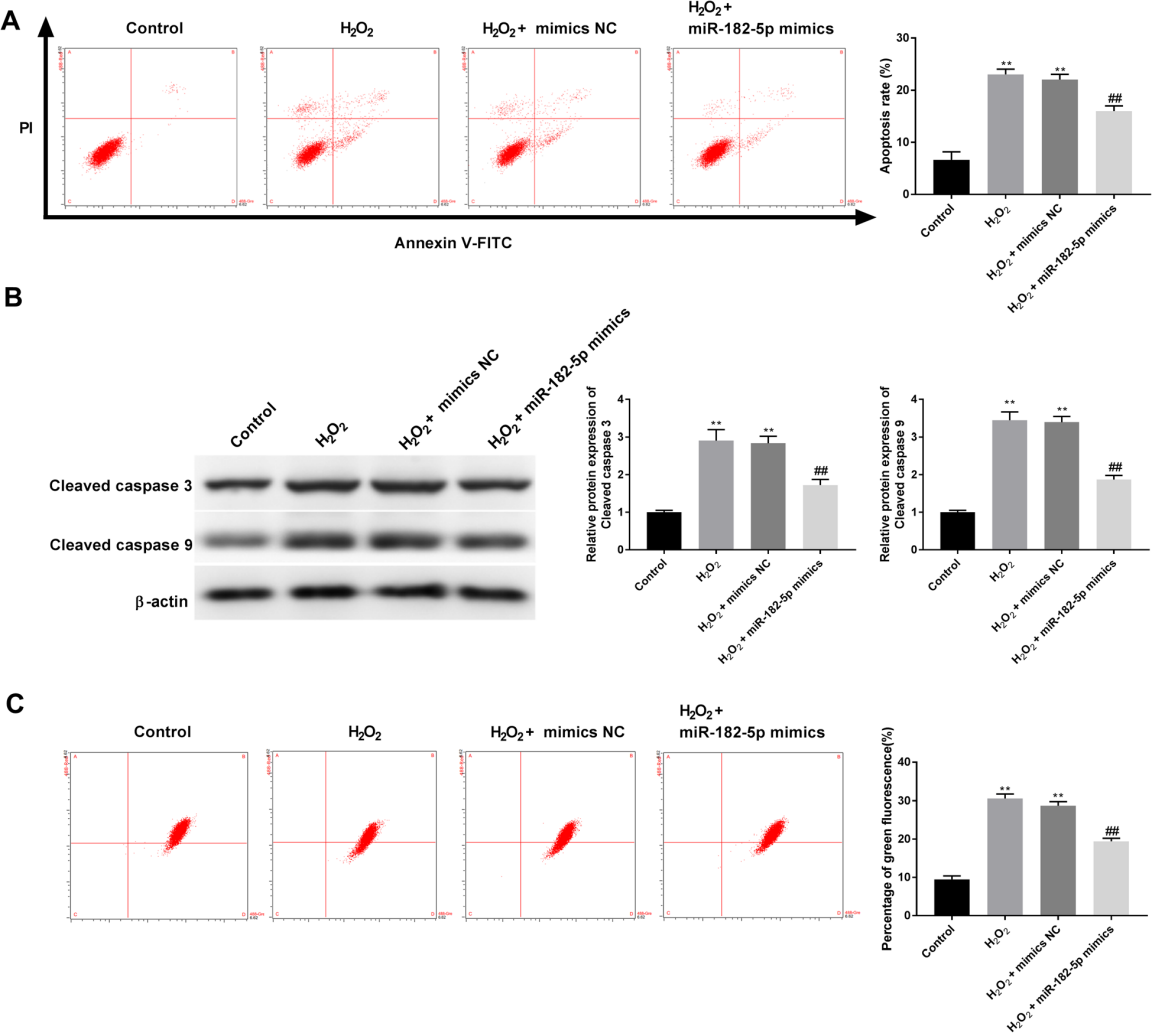
Overexpression of miR-182-5p protects HLE-B3 cells against oxidative stress-induced apoptosis. A. Cell apoptosis was examined by flow cytometric analysis. B. Expression of cleaved caspase 3 and cleaved caspase 9 in HLE-B3 cells was detected by western blot. C. The ratio of green/monomeric forms of JC-1 was calculated using flow cytometry. ** P < 0.01 vs. control group, ## P < 0.01 vs. H_2_O_2_ group. Independent experiments were carried out three times for each assay

Annexin V/PI double staining for detection of apoptosis revealed that miR-182-5p mimics reduced H_2_O_2_-induced apoptosis of HLE-B3 cells (Fig. 3A). Western blot results showed that H_2_O_2_ treatment up-regulated the expressions of cleaved caspase-3 and cleaved caspase-9, while their expressions were reversed with the transfection of miR-182-5p mimics (Fig. 3B). These results indicate that miR-182-5p mimics decreased the expression of pro-apoptotic proteins. To further investigate the mechanism underlying H_2_O_2_-induced apoptosis, the MMP of HLE-B3 cells was determined. After 12 h treatment with 250 μmol/L of H_2_O_2_, the MMP of HLE-B3 cells was significantly lower than that of control group cells, while the transfection of miR-182-5p mimics abolished the H_2_O_2_-induced decrease in MMP in HLE-B3 cells (Fig. 3C).

### Overexpression of miR-182-5p suppresses MAPK signalling in H_2_O_2_-treated HLE-B3 cells

**Fig. 4:**
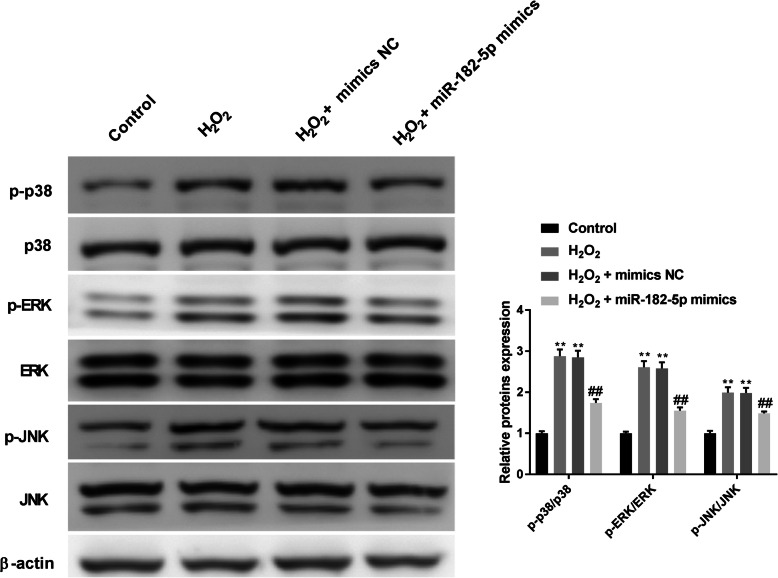
Overexpression of miR-182-5p suppresses p38 MAPK signalling in H_2_O_2_-treated HLE-B3 cells. Expressions of p-p-38, p-38, p-ERK, ERK, p-JNK and JNK were measured using western blot. ** P < 0.01 vs. control group, ## P < 0.01 vs. H_2_O_2_ group. Independent experiments were carried out three times for each assay

Our data showed that H_2_O_2_ treatment increased the levels of p-p38, p-ERK, and p-JNK. Pretreatment with miR-182-5p mimics apparently reversed the effect (Fig. 4). These data suggest that miR-182-5p suppressed MAPK signalling in HLE-B3 cells.

### MiR-182-5p binds NOX4 directly

**Fig. 5:**
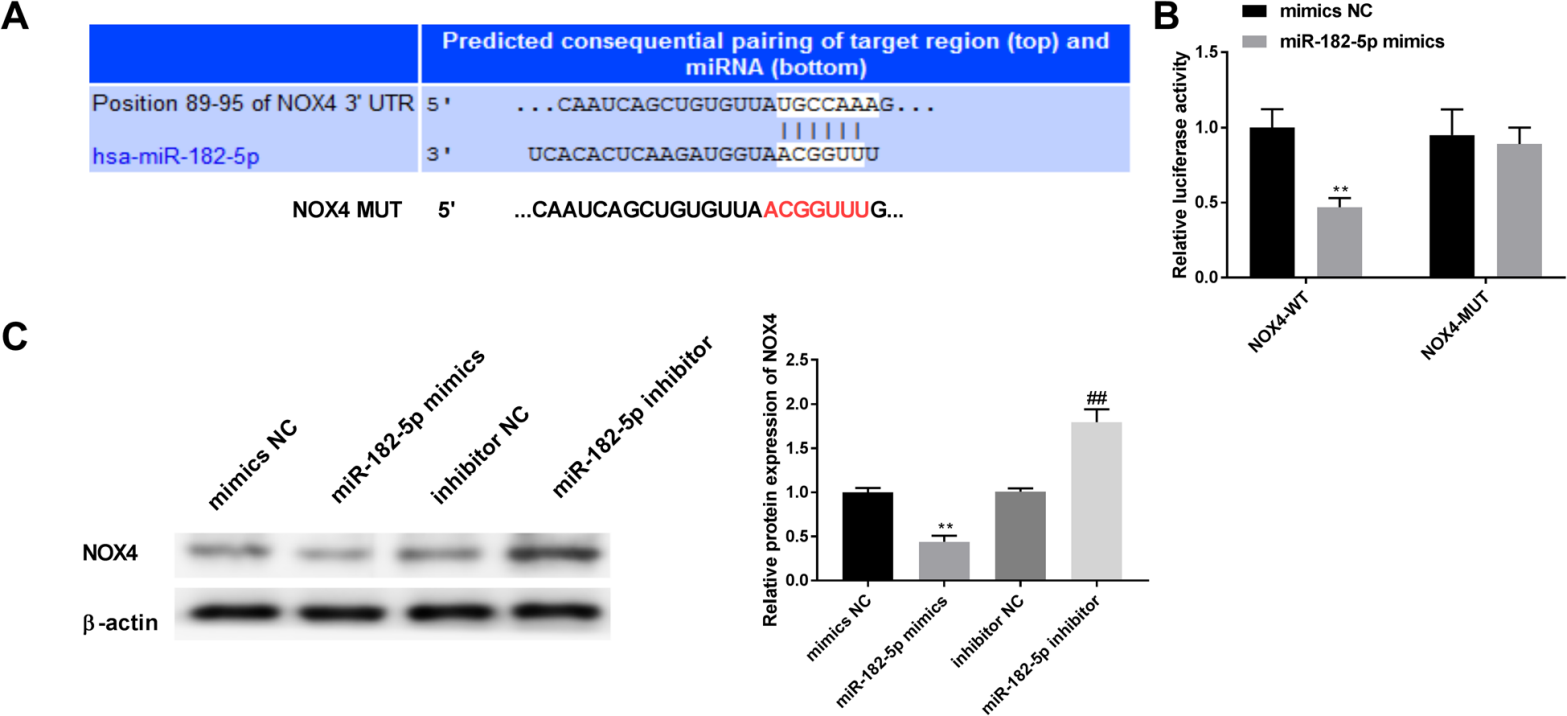
MiR-182-5p binds NOX4 directly. A. Putative miR-182-5p binding site in the 3’UTR region of NOX4. B. Luciferase activity of cells transfected with NOX4-WT, NOX4-MUT, mimics control and miR-182-5p mimics. ** P < 0.01 vs. NOX4-WT+ mimics NC group. C. The expression of NOX4 protein regulated by upregulated or downregulated miR-182-5p. ** P < 0.01 vs. mimics NC group, ## P < 0.01 vs. inhibitor NC group. Independent experiments were carried out three times for each assay

Through bioinformatics analysis using TargetScanHuman 7.2, we found that miR-182-5p could bind 3′UTR of NOX4 (Fig. 5A). Luciferase reporter assay showed that co-transfection of the wild type plasmid with miR-182-5p mimics suppressed luciferase reporter activity (Fig. 5B). We also detected NOX4 protein expression after transfection of miR-182-5p mimics or miR-182-5p inhibitor. NOX4 protein expression was significantly down-regulated by miR-182-5p mimics and up-regulated by miR-182-5p inhibitor (Fig. 5C). These results indicate that NOX4 directly targeted miR-182-5p.

### NOX4 overexpression reverses the protective effects of miR-182-5p mimics in H_2_O_2_-treated HLE-B3 cells

**Fig. 6:**
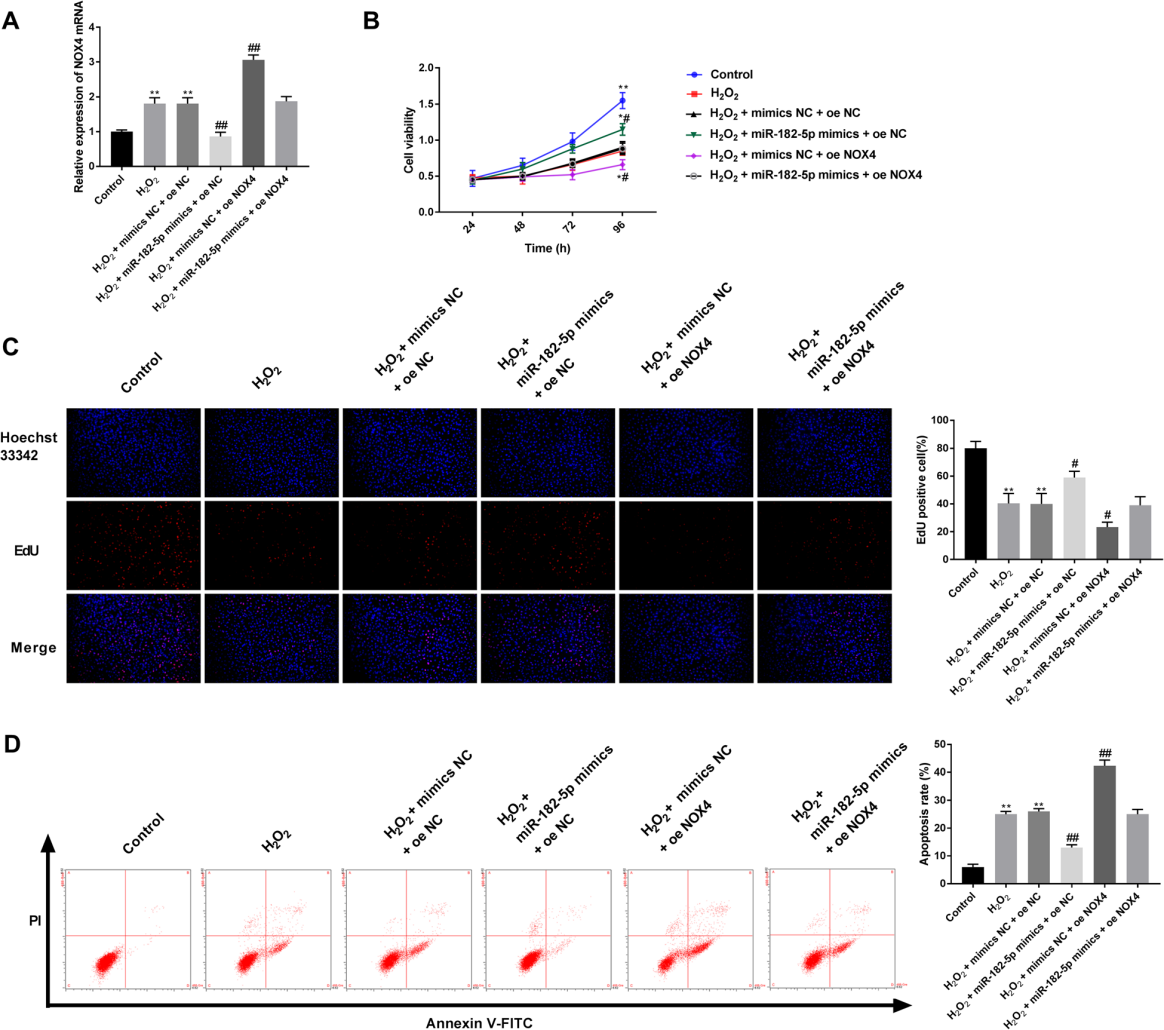
NOX4 overexpression reverses the protective effects of miR-182-5p mimics in H_2_O_2_-treated HLE-B3 cells. A. Expression of NOX4 was detected by qRT-PCR. B. HLE-B3 cell viability was detected by cell counting kit-8 assay. C. HLE-B3 cell proliferation was detected by EdU assay. D. Cell apoptosis was examined by flow cytometric analysis. ** P < 0.01 vs. control group, # P < 0.05 vs. H_2_O_2_ group, ## P < 0.01 vs. H_2_O_2_ group. Independent experiments were carried out three times for each assay

To determine whether NOX4 was related to the apoptosis of H_2_O_2_-induced HLE-B3 cells, oe-NOX4 or oe-NC was cotransfected with miR-182-5p mimics or negative controls into HLE-B3 cells. The transfection efficiency was determined using qRT-PCR. Compared with the control group, H_2_O_2_ treatment and oe-NOX4 increased the NOX4 expression, while miR-182-5p mimics decreased the NOX4 expression in the HLE-B3 cells. However, overexpression of NOX4 reversed this effect of miR-182-5p mimics (Fig. 6A). Moreover, NOX4 overexpression reversed the promotion of cell proliferation induced by miR-182-5p mimics in H_2_O_2_-treated HLE-B3 cells (Fig. 6B and C). The results also showed that NOX4 overexpression reversed the inhibition of apoptosis induced by miR-182-5p mimics in H_2_O_2_-treated HLE-B3 cells (Fig. 6D).

## Discussion

Oxidative stress is believed to take part in the pathogenesis of age-related cataract [15]. This study reported the protective effects of miR-182-5p in HLE-B3 cells against oxidative stress through inhibiting NOX4 expression and p38 MAPK pathway.

Accumulating evidence reveals that aberrant expression of miRNAs is observed after induction of oxidative stress. One study reported that miRNA-15a was significantly increased with the H_2_O_2_ exposure in HLE-B3 cells [16]. Another study demonstrated that the expression of miR-34a was up-regulated in HLE-B3 cells treated by H_2_O_2_ [17]. In this study, we observed that expression of miR-182-5p was significantly downregulated by the treatment of H_2_O_2_ in HLE-B3 cells, which was consistent with previous work [18]. Emerging evidence suggests that miR-182-5p contributes to anti-apoptotic and anti-oxidative processes. MiR-182-5p inhibits oxidative stress-induced apoptosis by targeting TLR4 [19]. In this article, miR-182-5p weakened apoptosis of H_2_O_2_-treated HLE-B3 cells by inhibiting the decline of MMP. The balance of MMP is important for maintaining the normal function of mitochondria. Thus, decreased MMP triggers mitochondrial swelling and rupturing of outer membrane, ultimately leading to apoptosis of cells [20, 21].

Oxidative stress particularly activates ERK, JNK, or p38 MAPK under different conditions [22–24]. Inhibition of p38 phosphorylation reduces H_2_O_2_-induced cellular apoptosis and inhibits ROS generation [24]. We found that miR-182-5p could suppress both p38 MAPK activation and ROS production in H_2_O_2_-treated HLE-B3 cells. Peng et al. also revealed that p-coumaric acid lessens H_2_O_2_-induced LECs apoptosis through suppressing phosphorylation of p-38, ERK, and JNK [25].

Prediction of target genes is a key step towards understanding the function of specific miRNAs. We found that miR-182-5p could bind the 3′UTR of NOX4 mRNA. Moreover, miR-182-5p mimics decreased the expression of NOX4 and miR-182-5p inhibitor increased the expression of NOX4. These results indicated that miR-182-5p may act via NOX4 to regulate cataract formation. NOX4 is a member of NOX family, which is the primary source of ROS [26]. NOX4-derived ROS play an important role in p38 MAPK signalling [27] and regulation of mitochondrial function [28]. A recent study reports that dapagliflozin decreases NOX4 levels in the LECs from fructose-fed rats, thereby reducing ROS generation during fructose-induced diabetic cataracts [29]. We confirmed that miR-182-5p inhibited H_2_O_2_-stimulated apoptosis of HLE-B3 cells; however, this effect was reversed by overexpression of NOX4. This is in accordance with previous findings that NOX4 reverses the protective effect of miR-423-5p in diabetic kidney diseases [30].

## Conclusion

In summary, we found that miR-182-5p alleviated H_2_O_2_-induced LECs injury. MiR-182-5p mediated its protective effects on LECs injury by directly targeting NOX4. Moreover, miR-182-5p decreased ROS production and p38 MAPK activity. Altogether, our results may provide novel insights for age-related cataract therapy.

## Data Availability

The datasets used and analyzed during the current study are available from the corresponding author on reasonable request.

## Abbreviations

CCK-8: Cell Counting Kit-8
DCFH-DA: 2′,7′: dichlorofluorescein diacetate
EMEM: Eagle’s minimum essential medium
EdU: 5-Ethynyl-2′-deoxyuridine
FBS: Fetal bovine serum
FITC: Fluorescein isothiocyanate
GSH-Px: Glutathione peroxidase
HLE-B3: Human lens epithelial B3
LECs: Lens epithelial cells
MAPK: Mitogen-activated protein kinase
MDA: Malondialdehyde
MFI: Mean fluorescent intensity
MiRNA: MicroRNA
MMP: Mitochondrial membrane potential
NOX4: Nicotinamide adenine dinucleotide phosphate oxidase subunit 4
PI: Propidium iodide
qRT-PCR: Quantitative real-time PCR
ROS: Reactive oxygen species
SOD: Superoxide dismutase

## References

[1] Liu YC, Wilkins M, Kim T, Malyugin B, Mehta JS (2017). Cataracts. Lancet.

[2] Khanna R, Pujari S, Sangwan V (2011). Cataract surgery in developing countries. Curr Opin Ophthalmol.

[3] Asbell PA, Dualan I, Mindel J, Brocks D, Ahmad M, Epstein S (2005). Age-related cataract. Lancet.

[4] Lansingh VC, Carter MJ, Martens M (2007). Global cost-effectiveness of cataract surgery. Ophthalmology.

[5] Watkinson S, Seewoodhary R (2015). Cataract management: effect on patients' quality of life. Nurs Stand.

[6] Song P, Wang H, Theodoratou E, Chan KY, Rudan I (2018). The national and subnational prevalence of cataract and cataract blindness in China: a systematic review and meta-analysis. J Glob Health.

[7] Li G, Song H, Chen L, Yang W, Nan K, Lu P (2017). TUG1 promotes lens epithelial cell apoptosis by regulating miR-421/caspase-3 axis in age-related cataract. Exp Cell Res.

[8] Beebe DC, Holekamp NM, Shui YB (2010). Oxidative damage and the prevention of age-related cataracts. Ophthalmic Res.

[9] Filipowicz W, Bhattacharyya SN, Sonenberg N (2008). Mechanisms of post-transcriptional regulation by microRNAs: are the answers in sight?. Nat Rev Genet.

[10] Icme G, Yilmaz A, Dinc E, Gorur A, Fidanci SB, Tamer L (2019). Assessment of miR-182, miR-183, miR-184, and miR-221 expressions in primary Pterygium and comparison with the Normal conjunctiva. Eye Contact Lens.

[11] Liu Y, Bailey JC, Helwa I, Dismuke WM, Cai J, Drewry M, Brilliant MH, Budenz DL, Christen WG, Chasman DI (2016). A common variant in MIR182 is associated with primary open-angle Glaucoma in the NEIGHBORHOOD consortium. Invest Ophthalmol Vis Sci.

[12] Wu CR, Ye M, Qin L, Yin Y, Pei C (2017). Expression of lens-related microRNAs in transparent infant lenses and congenital cataract. Int J Ophthalmol.

[13] Huang YX, Nie XG, Li GD, Fan DS, Song LL, Zhang XL (2018). Downregulation of microRNA182 inhibits cell viability, invasion and angiogenesis in retinoblastoma through inhibition of the PI3K/AKT pathway and CADM2 upregulation. Int J Oncol.

[14] Szemraj M, Oszajca K, Szemraj J, Jurowski P (2017). MicroRNA expression analysis in serum of patients with congenital hemochromatosis and age-related macular degeneration (AMD). Med Sci Monit.

[15] Periyasamy P, Shinohara T (2017). Age-related cataracts: role of unfolded protein response, Ca(2+) mobilization, epigenetic DNA modifications, and loss of Nrf2/Keap1 dependent cytoprotection. Prog Retin Eye Res.

[16] Li Q, Pan H, Liu Q. MicroRNA-15a modulates lens epithelial cells apoptosis and proliferation through targeting B-cell lymphoma-2 and E2F transcription factor 3 in age-related cataracts. Biosci Rep 2019;39(12). 10.1042/BSR20191773 PMC6900469 31737898

[17] Li QL, Zhang HY, Qin YJ, Meng QL, Yao XL, Guo HK (2016). MicroRNA-34a promoting apoptosis of human lens epithelial cells through down-regulation of B-cell lymphoma-2 and silent information regulator. Int J Ophthalmol.

[18] Li X, Wang Q, Ren Y, Wang X, Cheng H, Yang H, Wang B (2019). Tetramethylpyrazine protects retinal ganglion cells against H2O2induced damage via the microRNA182/mitochondrial pathway. Int J Mol Med.

[19] Qin SB, Peng DY, Lu JM, Ke ZP (2018). MiR-182-5p inhibited oxidative stress and apoptosis triggered by oxidized low-density lipoprotein via targeting toll-like receptor 4. J Cell Physiol.

[20] Estaquier J, Vallette F, Vayssiere JL, Mignotte B (2012). The mitochondrial pathways of apoptosis. Adv Exp Med Biol.

[21] Kim GT, Lee SH, Kim YM (2016). Torilis japonica extract-generated intracellular ROS induces apoptosis by reducing the mitochondrial membrane potential via regulation of the AMPK-p38 MAPK signaling pathway in HCT116 colon cancer. Int J Oncol.

[22] Waetzig GH, Seegert D, Rosenstiel P, Nikolaus S, Schreiber S (2002). p38 mitogen-activated protein kinase is activated and linked to TNF-alpha signaling in inflammatory bowel disease. J Immunol.

[23] Hua X, Chi W, Su L, Li J, Zhang Z, Yuan X (2017). ROS-induced oxidative injury involved in pathogenesis of fungal keratitis via p38 MAPK activation. Sci Rep.

[24] Bai J, Zheng Y, Dong L, Cai X, Wang G, Liu P (2015). Inhibition of p38 mitogen-activated protein kinase phosphorylation decreases H(2)O(2)-induced apoptosis in human lens epithelial cells. Graefes Arch Clin Exp Ophthalmol.

[25] Peng J, Zheng TT, Liang Y, Duan LF, Zhang YD, Wang LJ, He GM, Xiao HT. p-Coumaric Acid Protects Human Lens Epithelial Cells against Oxidative Stress-Induced Apoptosis by MAPK Signaling. Oxidative Med Cell Longev 2018;10(8549052). 10.1155/2018/8549052 PMC5914090 29849919

[26] Bedard K, Krause KH (2007). The NOX family of ROS-generating NADPH oxidases: physiology and pathophysiology. Physiol Rev.

[27] Huang Y, Cai GQ, Peng JP, Shen C (2018). Glucocorticoids induce apoptosis and matrix metalloproteinase-13 expression in chondrocytes through the NOX4/ROS/p38 MAPK pathway. J Steroid Biochem Mol Biol.

[28] Li Z, Deng W, Cao A, Zang Y, Wang Y, Wang H, Wang L, Peng W (2019). Huangqi decoction inhibits hyperglycemia-induced podocyte apoptosis by down-regulated Nox4/p53/Bax signaling in vitro and in vivo. Am J Transl Res.

[29] Chen YY, Wu TT, Ho CY, Yeh TC, Sun GC, Kung YH, Wong TY, Tseng CJ, Cheng PW. Dapagliflozin Prevents NOX- and SGLT2-Dependent Oxidative Stress in Lens Cells Exposed to Fructose-Induced Diabetes Mellitus. Int J Mol Sci 2019;20(18). 10.3390/ijms20184357 PMC6770809 31491943

[30] Xu Y, Zhang J, Fan L, He X (2018). miR-423-5p suppresses high-glucose-induced podocyte injury by targeting Nox4. Biochem Biophys Res Commun.

